# Evolutionary analysis of the Dof family in plants and identification of its members in tobacco

**DOI:** 10.3389/fpls.2026.1809808

**Published:** 2026-04-28

**Authors:** Bolun Zhi, Hao Chen, Xuebo Wang, Chuanliang Liu, Jiqin Li, Zhuwen Ma, Weicai Zhao, Zhenrui Huang, Xiaoying Pan

**Affiliations:** 1 Guangdong Key Laboratory for Crops Genetic Improvement, Crops Research Institute, Guangdong Academy of Agricultural Sciences (GAAS), Guangdong Provincial Engineering & Technology Research Center for Tobacco Breeding and Comprehensive Utilization, Guangzhou, China; 2 College of Agronomy, South China Agricultural University, Guangzhou, China; 3 Tobacco Science Research Institute of Guangdong Province, Shaoguan, China

**Keywords:** CDF subfamily, cold stress, DOF transcription factor, phylogenetic analysis, tobacco

## Abstract

DNA-binding with One Finger (Dof) transcription factors are plant-specific regulators involved in diverse biological processes, including growth, development, and stress responses. However, the Dof family in tobacco (*Nicotiana tabacum* L.) remains poorly characterized. Here, we performed a genome-wide identification and comprehensive analysis of *Dof* genes in tobacco, revealing 75 *NtDof* members. Through phylogenetic reconstruction, gene structure analysis, and cis-regulatory element prediction, we classified these genes into seven subfamilies and found that the CDF clade exhibits conserved exon–intron structures, unique motif compositions, and enrichment of stress- and hormone-responsive cis-elements. Expression profiling showed that most *NtCDF* genes respond dynamically to cold and magnesium deficiency stresses. Virus-induced gene silencing (VIGS) of two representative *NtCDF* genes, *NtDof40* and *NtDof63*, revealed their opposing roles in cold tolerance: *NtDof40* acts as a positive regulator, while *NtDof63* functions as a negative regulator. Our findings provide a comprehensive evolutionary framework for the Dof family in tobacco and highlight the functional divergence of CDF subfamily members in abiotic stress adaptation, offering potential targets for stress-resistance breeding.

## Introduction

Transcription factors (TFs) have been demonstrated to participate in plant stress responses, many operating through sophisticated regulatory networks (Manna et al., 2021). Different TFs recognize distinct genomic domains to execute specific functions, while their functional relationships are primarily established through protein-protein interactions (Bai et al., 2025; Bhatt et al., 2025; Song et al., 2026). Most derive from multigene families that have undergone repeated duplication events during land plant evolution (Panchy et al., 2016). Given the pivotal regulatory roles of TFs in evolutionary adaptation and functional expression, investigating their mechanisms is crucial for understanding and exploiting species’ genetic potential (Singh, 1998; Jakoby et al., 2002). Ideally, such investigations should initiate from genome-wide expression profiling (Riechmann et al., 2000).

DNA-binding with one finger (Dof) proteins are a group of plant-specific TFs initially identified in maize (Yanagisawa and Izui, 1993; Yanagisawa, 2004; Waschburger et al., 2023). The *Dof* gene family maintains relatively small sizes in plant genomes but shows substantial variation in gene copy numbers among different species (Zou and Sun, 2023). Phylogenetic classification of *DOF* proteins from *Arabidopsis*, rice, barley and *Brachypodium* based on C-terminal regulatory domains and exon-intron architectures revealed four distinct subfamilies (Hernando-Amado et al., 2012). The Cycling *DOF* Factors (*CDF*) clade clustered within subfamily D and demonstrated photoperiod-dependent transcriptional regulation (Imaizumi et al., 2005). *Dof* transcription factors modulate diverse physiological processes by regulating gene expression, including seed germination (Da Silva et al., 2016), floral transition (Zhang et al., 2019), root system development (Rymen et al., 2017; Fan et al., 2025), light signaling transduction (Yang et al., 2010), and phytohormone responses (Zhuo et al., 2020). The *CDF* subfamily has evolved specialized functions in stress adaptation, playing regulatory roles in drought resistance (Chen et al., 2020b), salinity tolerance (Renau-Morata et al., 2017), freezing tolerance (Xu and Dai, 2016), and pathogen defense through distinct molecular mechanisms.

Tobacco (*Nicotiana tabacum* L.) is a commercially important leaf crop on a global scale; however, its productivity is significantly susceptible to abiotic stressors, notably chilling stress, drought, salinity, and extreme heat (Liu et al., 2024). Mounting pressure from climate disruptions has elevated the urgency for research into stress-resilient varieties (Kopeć, 2024). Identifying novel tolerance genes represents a critical pathway for both deciphering fundamental plant adaptation mechanisms and securing sustainable tobacco production through climate-smart agriculture. Although the *Dof* family plays well-documented roles in regulating both plant growth and stress responses, current research on this transcription factor family remains at a foundational stage, with functional characterization in tobacco particularly lacking (Zou and Sun, 2023). The present study conducted comprehensive characterization of the *NtDof*, including gene identification, evolutionary analysis, phylogenetic reconstruction, and structural examination. We further employed whole-transcriptome data to analyze the expression profiles of *Dof* genes under various stress conditions. Specifically, we functionally characterized two selected *CDF* genes during cold stress through enzymatic activity assays to delineate their regulatory pathways. These findings establish fundamental knowledge about this important gene family in tobacco and provide a critical foundation for future functional studies and the development of stress-resistant tobacco cultivars.

## Materials and methods

### Evolutionary identification and comparative analysis of *Dof* gene family

To obtain a comprehensive and evolutionarily conserved set of Dof protein sequences, *Arabidopsis thaliana* Dof (*AtDof)* proteins were used as query sequences (Yanagisawa, 2002). BLASTp searches (E-value < 1×10^-10^) were performed against annotated and predicted protein sequences datasets from 11 representative plant species covering major evolutionary lineages, using the Phytozome and NCBI databases. These species included three ferns (*Ceratopteris thalictroides*, *Selaginella tamariscina*, *Diphasiastrum complanatum)* (Banks et al., 2011; Marchant et al., 2022; Xu et al., 2024), three gymnosperms (*Cycas revoluta Thunb*, *Thuja plicata*, *Ginkgo biloba*) (Gu et al., 2022; Liu et al., 2022; Shalev et al., 2022), and five angiosperms (*Amborella trichopoda*, *Solanum lycopersicum*, *Arabidopsis thaliana*, *Zea mays*, and *Triticum aestivum*) (Amborella Genome Project, 2013; Hufford et al., 2021; Zhu et al., 2021; Zhou et al., 2022). Candidate sequences underwent additional filtering to retain only those that contained a complete C2-C2 zinc finger domain and conserved DNA-binding motifs. Sequences with incomplete annotations, low quality, fragmented structures, poly-N regions, or those identified as pseudogenes were excluded from further analysis. Following manual curation and quality control, a total of 327 high-confidence *Dof* genes were selected for phylogenetic tree construction.

Protein sequences are provided in **Supplementary Table 1**.

### Genome wide identification of *Dof* genes in tobacco

*Dof* proteins in tobacco were identified by using a Hidden Markov Model (HMM) search based on the Dof domain profile (PF02701) obtained from the Pfam database (https://pfam.xfam.org/). In parallel, BLAST searches, (e-value < 1 × 10^−10^) were conducted against the tobacco reference genome protein sequences. All candidate Dof proteins were further validated by Conserved Domain Data (CDD) analysis (https://www.ncbi.nlm.nih.gov/Structure/bwrpsb/bwrpsb.cgi) to confirm the presence of the conserved Dof DNA binding domain.

### Phylogenetic reconstruction and gene structural analysis of *NtDof* genes

Multiple sequences alignment and phylogenetic analyses were performed using MEGA11 (Pennsylvania, USA). A phylogenetic tree was constructed using the maximum likelihood (ML) method with default parameters. Gene structure and conserved domain organization of *NtDof* genes were visualized using TBtools (Chen et al., 2020a). Information on exon-intron organization was extracted from the tobacco genome (Wang et al., 2024).

### Conserved motif identification, chromosomal distribution and gene duplication analysis

Conserved motifs within NtDof proteins were identified using MEME (https://meme-suite.org/meme/tools/meme) with default settings. The chromosomal localization information for *NtDof* genes was obtained from the tobacco gene model annotation files NtSR1 (http://lifenglab.hzau.edu.cn/Nicomics/). To investigate the gene duplication events, full length NtDof protein sequences were subjected to BLAST analysis, and duplication relationships were detected using Mcscan X (e-value 1 × 10^−5^, considering syntenic blocks and nearby gene loci).

### Promoter cis-acting regulatory element analysis

The promoter sequences of *NtDof* genes were retrieved from the tobacco genome database. The 2.0 kb region upstream regions from the translation start site (ATG) were analyzed Using Plantcare (https://bioinformatics.psb.ugent.be/webtools/plantcare/html/), to identify putative cis-acting regulatory elements involved in transcriptional regulation.

### Expression profiling of *NtDof* genes based on transcriptome data

Transcriptome datasets were used to investigate the spatial and temporal expression patterns of *NtDof* genes. RNA-seq data generated in our laboratory from a previous study were re-analyzed to quantify the expression levels of *NtDof* genes across different tissues and developmental stages (accession number: PRJNA1093408, PRJNA1093408, PRJNA1223488). Normalized expression values were used to generate heatmaps with hierarchical clustering using TBtools, allowing visualization of expression divergence and co-expression patterns among *NtDof* family members.

### Quantitative real-time polymerase chain reaction

Total RNA of high-quality was extracted from tobacco samples and reverse transcribed in to first strand cDNA using the HiScript II 1st Strand cDNA Synthesis Kit (Vazyme, Nanjing, China) following the manufacturer’s instructions. The gene specific primers were designed using Primer5.0 software. The qRT-PCR assays were performed using a CFX96 real-time PCR detection system (Bio-Rad, CA, USA). Each 10 μL reaction mixture contained 1 μL cDNA template, 0.25 μL of each primer, 5 μL SYBR Green Mix, and 3.5 μL ddH_2_O. The thermal cycling conditions were as follows: 95 °C for 30 s, 40 cycles of 95 °C for 5 s, and 60 °C for 30 s. Relative gene expression levels were calculated using the (Ct) 2^-ΔΔCt^ method, with *NtACTIN* as the internal reference gene. Each experiment was performed with three biological replicates and three technical replicates.

### Virus induced gene silencing of the candidate genes

Virus-induced gene silencing (VIGS) was employed to investigate the functional roles of target *NtCDF* genes. Based on the online website VIGS tool (https://vigs.solgenomics.net/), specific primers were designed to amplify a 300 bp silencing fragment using the tobacco seedling root cDNA as a template. The amplified fragments were initially cloned into the pCE2 TA/Blunt-Zero Vector (Vazyme Biotech, Nanjing, China) and positive clones were confirmed by sequencing. The verified silencing fragments were subsequently inserted into the linearized pTRV2 vector via the homologous recombination using *EcoR*I and *Kpn*I restriction endonucleases. Recombinant plasmids were confirmed by sequencing. Agrobacterium cultures harboring pTRV2-empty vector, pTRV2-*PDS*, and pTRV2-*NtCDFs* were each mixed with the pTRV1 vector at a 1:1 volume ratio. The resulting agrobacterium mixtures were infiltrated into tobacco seedlings and incubated in a light controlled growth chamber for approximately 10 days. After the incubation period, the gene silencing efficiency of *NtCDFs* was assessed by expression analysis. All experimental procedures were performed in accordance with the methodologies established in previous studies (Chen et al., 2024). The primer sequences are listed in **Supplementary Table 2**.

### Determination of physiological and biochemical parameters

Relative conductivity assay was determined following previously published protocols (Chen et al., 2024). The levels of malondialdehyde (MDA) and the enzymatic activities of catalase (CAT), superoxide dismutase (SOD), and peroxidase (POD) were determined using assay kits (Solarbio, China).

## Results

### Evolutionary phylogenomics of the plant Dof gene family

**Figure 1:**
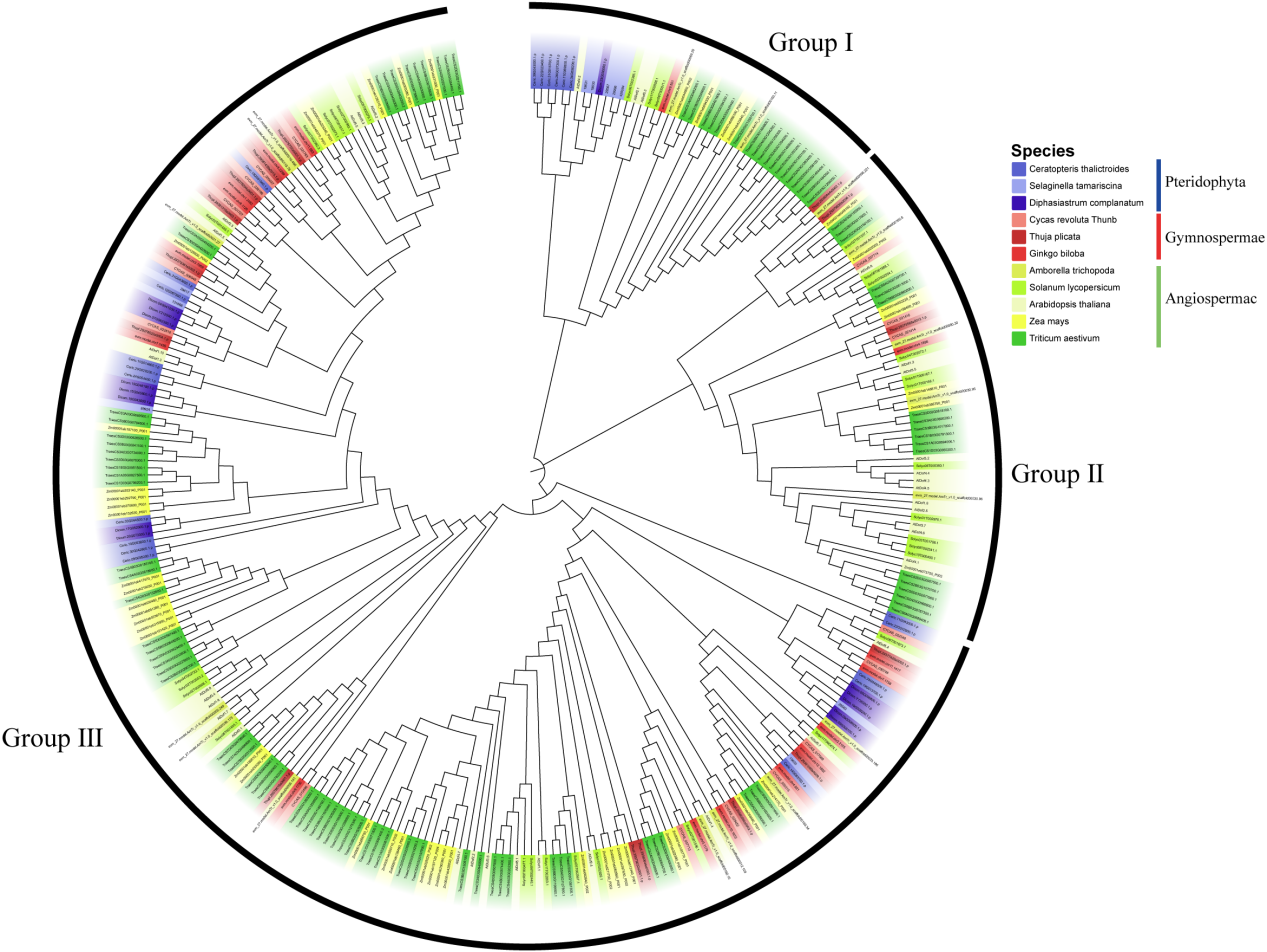
A phylogenetic tree was constructed using *Dof* protein sequences from 11 species representing distinct evolutionary stages, ranging from pteridophyta to angiosperms.

In order to systematically examine the cross-species evolutionary patterns of the *Dof* gene family, we selected 11 representative species and performed phylogenetic tree construction along with related analyses on the *Dof* gene family members of these species. Phylogenetic reconstruction revealed that the plant *Dof* gene family can be classified into three distinct subfamilies (**Figure 1**). The topology of the phylogenetic tree suggests that the *Dof* gene family originated early during plant evolution and underwent initial major functional diversification before the emergence of seed plants. The greatest abundance of *Dof* genes is observed in angiosperms, indicating that this gene family underwent multiple rounds of gene duplication and functional specialization throughout angiosperm evolution (**Figure 1**). This phenomenon is likely intricately linked to the adaptation of angiosperms to diverse environments and the evolution of complex traits.

**Figure 2:**
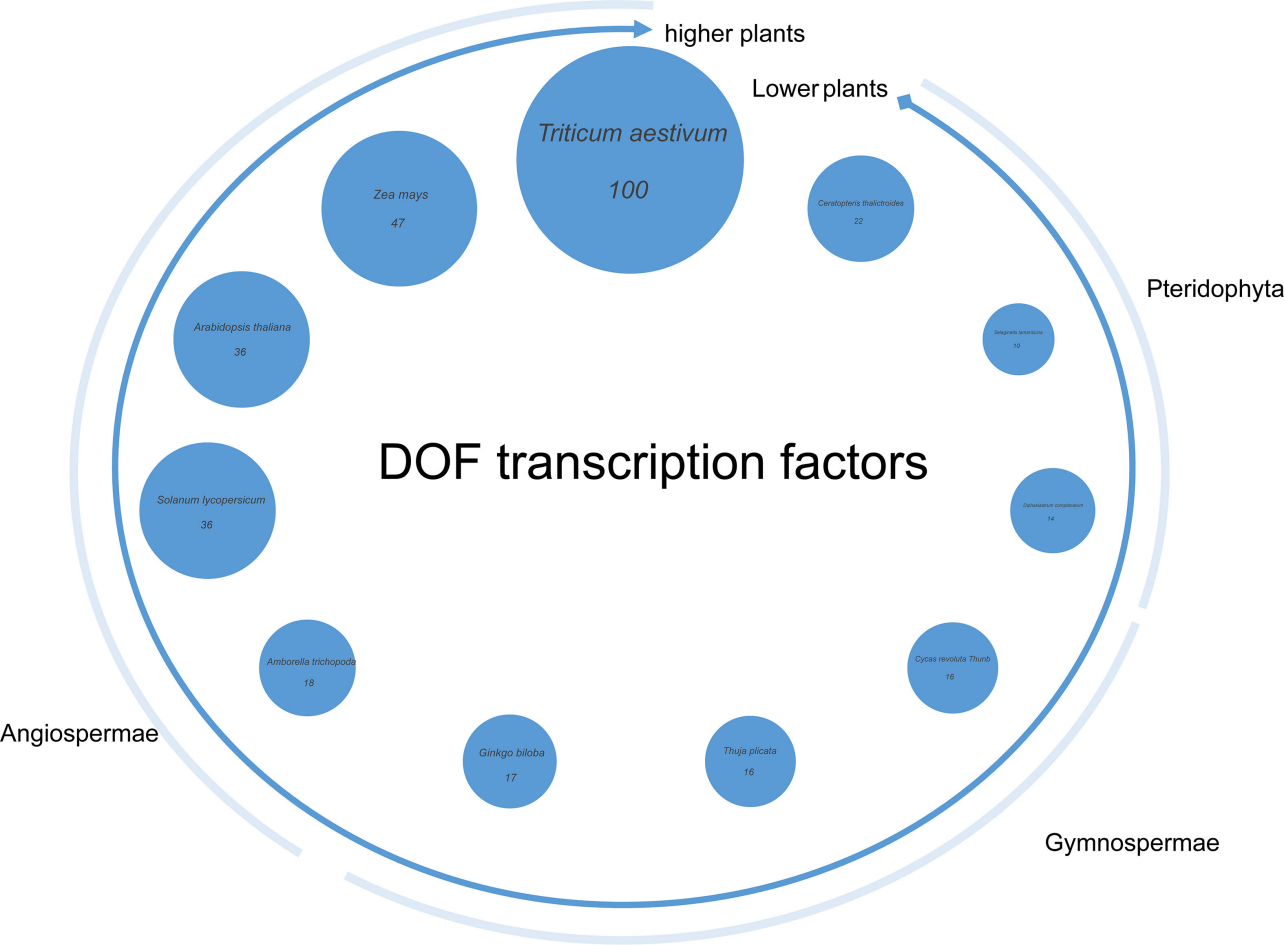
The quantitative relationship of *Dof* gene family members contained in 11 species from pteridophyta to angiosperms.

To elucidate the evolutionary expansion patterns of the *Dof* transcription factor family in plants, a quantitative comparison of *Dof* gene numbers across representative plant species was performed and visualized using bubble charts. The results revealed significant variation in the size of the *Dof* gene family among different plant taxa, showing a strong correlation with their evolutionary status. Among lower plants, ferns contained the smallest number of *Dof* genes, ranging from 10 to 22. Gymnosperms possess between 16 and 17 *Dof* genes. In contrast, higher plants demonstrated a marked expansion, particularly in monocotyledons. For instance, common *Triticum aestivum* contains 100 *Dof* genes, which is seven times the number found in *Selaginella tamariscina*, while *Zea mays* contained 47 *Dof* genes. Even dicotyledonous species, such as *Arabidopsis thaliana* and *Solanum lycopersicum*, each possess 36 *Dof* genes. Notably, *Amborella trichopoda*, the most primitive extant angiosperm, contains only 18 *Dof* genes, a number more closely aligned with gymnosperms (**Figure 2**). This suggests that the extensive gene expansion observed in angiosperms occurred subsequent to their divergence into dicotyledons and monocotyledons.

### Functional classification of *Dof* transcription factors

To gain deeper insights into the functional diversity of *Dof* TFs, we characterized the *Dof* family members using the NCBI and TAIR (https://www.arabidopsis.org/) databases. Based on the biological roles, *Dof* family genes were categorized into five functional categories: hormone responsive, stress responsive, signal transduction, growth and development related and functionally uncharacterized groups. Currently, known *Dof* family members are predominantly associated with plant growth and development functions, while very few members specifically *AtDof3.3*, *AtDof4.6*, and *AtDof5.8* have been reported to participate in stress responses (**Supplementary Table 3**). The majority of these characterized *AtDofs* family contributes to growth and development primarily by influencing the plant vascular system and seed development. The *AtDof* family members may also play key roles in cellular division processes within the vascular system. These findings provide a valuable reference framework for understanding the functions of *Dof* family members across diverse plant species.

### Identification and phylogenetic classification of *Dof* genes in tobacco

**Figure 3:**
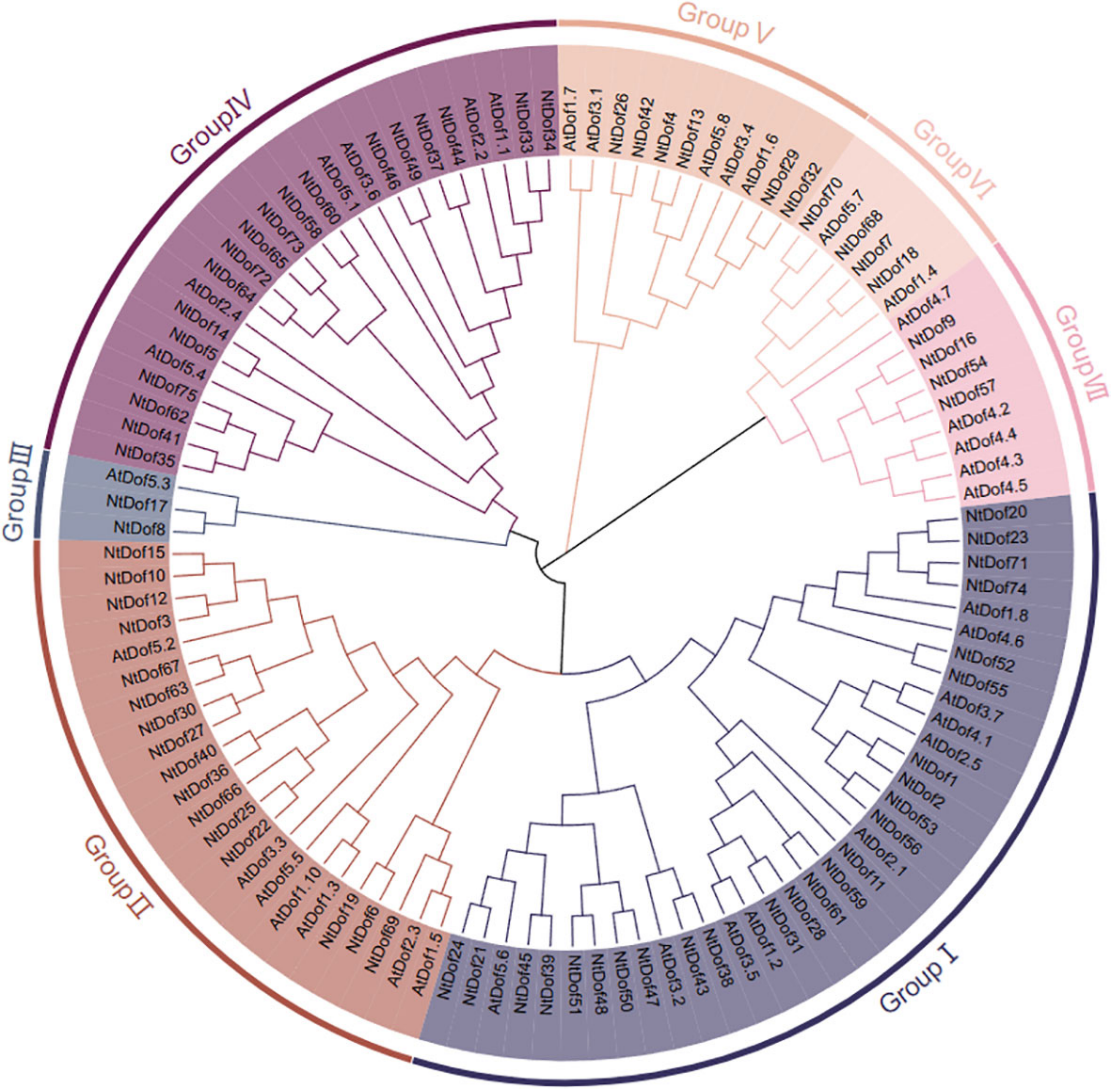
Phylogenetic analysis of the *Dof* genes in tobacco and Arabidopsis.

In order to investigate the phylogenetic relationship between tobacco and *Arabidopsis thaliana*, a comprehensive analysis was conducted in which 75 *Dof* genes (*NtDof1*–*NtDof75*) were identified in tobacco (**Supplementary Table 4**). These genes were subsequently subjected to phylogenetic analysis alongside 36 *Dof* genes from *Arabidopsis*. The analysis revealed that the *Dof* genes are categorized into seven subfamilies (GroupI-GroupVII) (**Figure 3**). Among these Group I represents the largest subfamily, with 35 members, with 25 *NtDofs* and 10 *AtDofs*. In contrast, Group III was the smallest containing only three members, of which two are from tobacco and one from *Arabidopsis*. The uneven distribution of tobacco and *Arabidopsis Dof* genes among different subfamilies suggests potential divergence in gene retention and expansion, implying that distinct *Dof* subfamilies may have undergone functional specialization during evolution.

### Gene structure, conserved motifs, chromosomal distribution, and duplication patterns of tobacco *NtDof* genes

**Figure 4:**
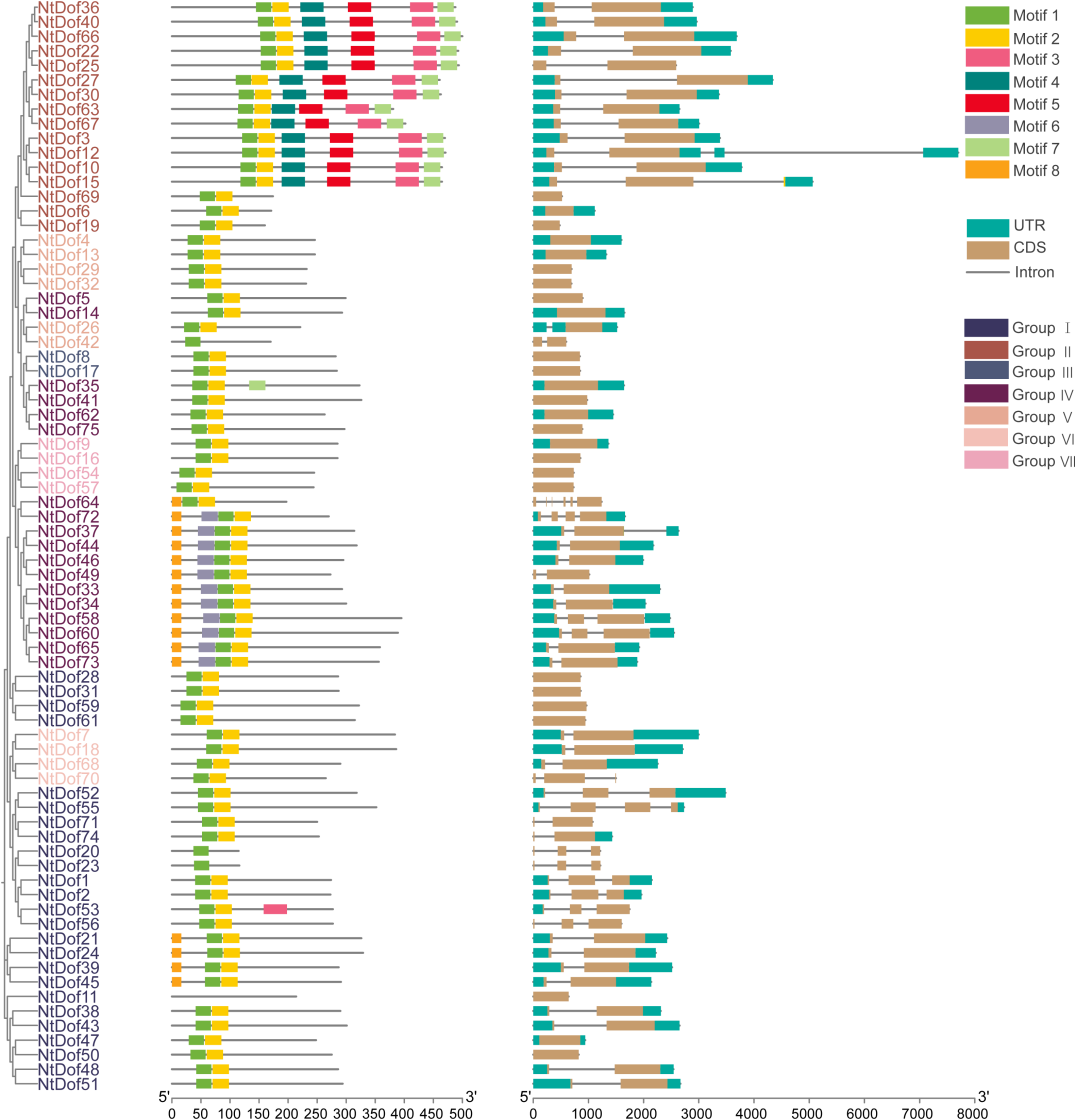
Characterization of tobacco *Dof* genes. Conserved motif distribution in the *Dof* proteins. Tobacco *Dof* gene exon/intron structure. CDs are represented as light gray boxes. Introns are shown by black lines, whereas untranslated regions (UTRs) are represented by teal boxes.

Gene structural variation is a critical driver of gene family diversification and functional differentiation. To explore the structural features of tobacco *Dof* genes, the exon-intron organization of all 75 *NtDof* genes was analyzed using TBTools. Within the *NtCDF* subfamily (Group II), all members, except *NtDof6*, *NtDof19*, and *NtDof69*, exhibited a conserved gene structure consisting of two exons separated by a single intron indicating a high degree of structural conservation within the clade (**Figure 4**). To further elucidate functional conservation and divergence, conserved motif analysis was performed using MEME. Eight distinct conserved motifs, ranging from 17 to 41 amino acids in length, were identified across the 75 *NtDofs* (**Supplementary Table 5**). Motif 1 was consistently found in all NtDof proteins, aligning with its function as the core Dof DNA-binding domain. Notably, members of the *NtCDF* subfamily (Group II), with the exception of *NtDof6*, *NtDof19*, and *NtDof69*, uniquely possessed motifs 3, 4, 5, and 7. This subfamily specific motif composition implies that the *NtCDF* clade may have developed lineage specific functional modules that are not present in other NtDof subfamilies (**Figure 4**).

**Figure 5:**
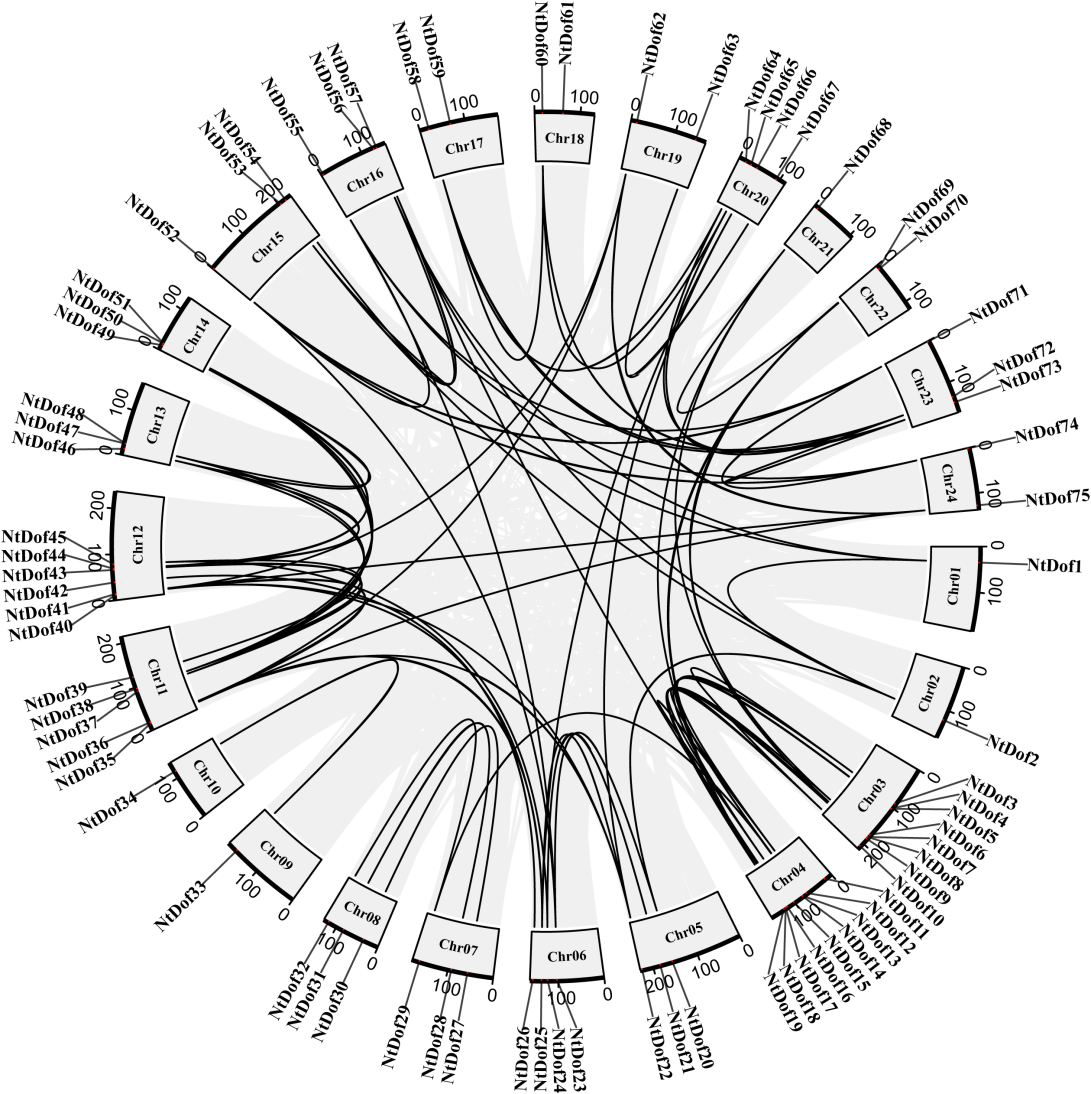
Chromosomal distribution and gene duplication patterns of tobacco *Dof* genes, with chromosomes 1–24 displayed in a circular layout and black curves indicating the duplication relationships among *NtDof* genes.

Chromosomal localization analysis demonstrated that the 75 *NtDof* genes are unevenly distributed across all 24 tobacco chromosomes. Chromosome 4 harbored the highest number of *NtDof* members (9), whereas Chromosomes 1, 9, 10 and 21 each contained only one member (**Figure 5**). Gene duplication analysis indicated that all *NtDofs*, except *NtDof59* and *NtDof61*, originated from duplication events. Among these, tandem duplications (occurring between adjacent chromosomal regions) were predominant, while segmental duplications (occurring across distant chromosomes) were comparatively infrequent. This distribution pattern suggests that tandem duplication likely served as the primary mechanism facilitating the expansion of the *NtDof* gene family in tobacco (**Figure 5**).

### Analysis of *cis*-acting regulatory elements in *NtDof* promoters

**Figure 6:**
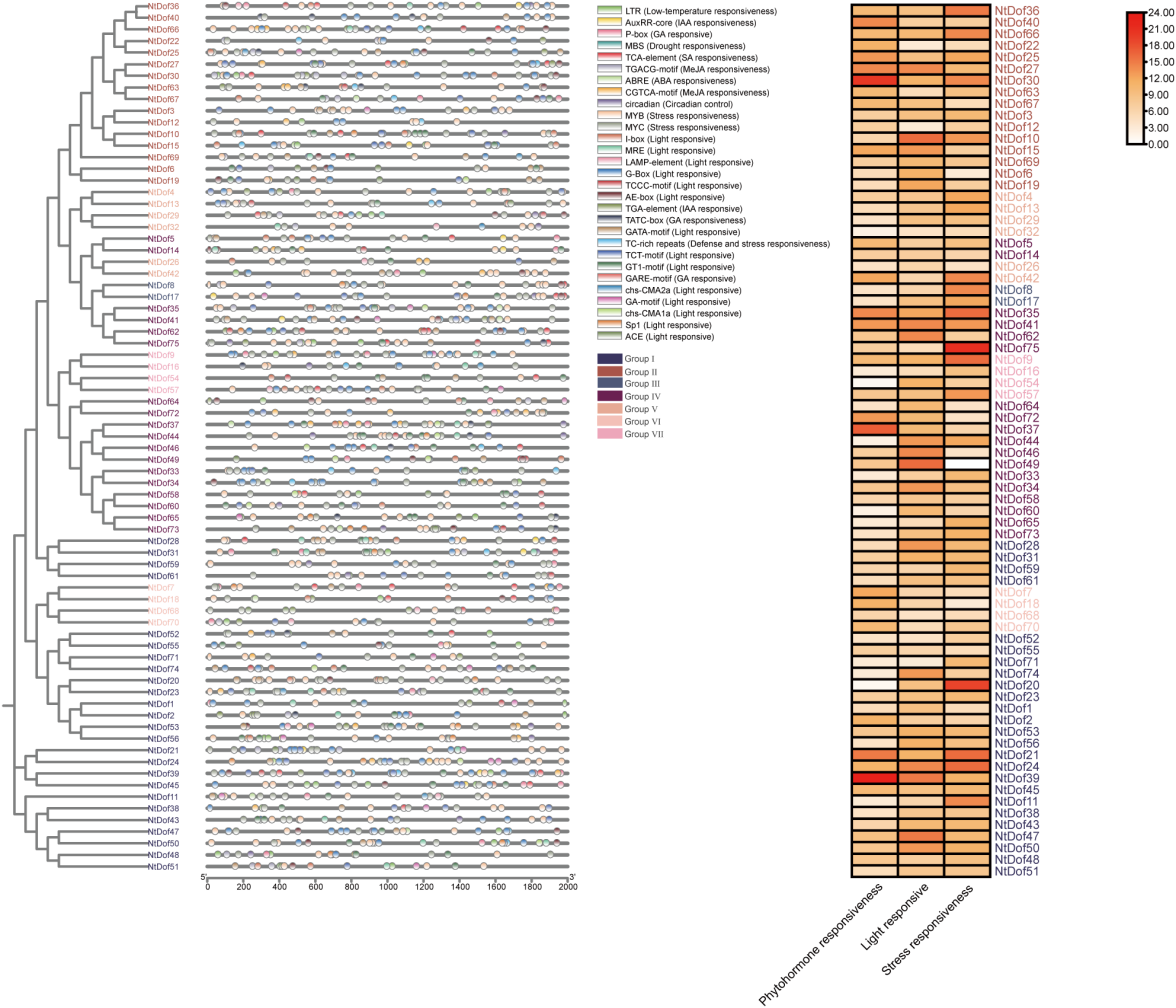
Schematic representation of cis-elements detected in *NtDof* gene promoter.

To elucidate the regulatory mechanisms underlying *NtDof* genes, we conducted an analysis of cis-acting elements located within the 2kb upstream region of the ATG start codon for each gene, utilizing the PlantCARE database. In addition to identifying core expression elements such as CAAT and TATA boxes, we discovered a variety of hormone responsive elements, including those responsive to abscisic acid (ABA), methyl jasmonate (MeJA), and gibberellins (GA), as well as elements associated with stress responses, such as those related to drought and low temperature response (LTR) were also identified (**Supplementary Table 6**). By categorizing these elements into hormone, light, and stress responsive categories, heatmap visualization indicated that members of the *NtCDF* subfamily exhibited a higher enrichment of stress and hormone responsive elements compared to other *NtDof* members (**Figure 6**). When integrated with previous functional studies of *NtCDF* genes, these findings suggest that *NtCDF* gene family members may possess species specific regulatory and functional characteristics that differentiate them from homologous *Dof* genes in other plant species.

### Expression profiles of *NtCDF* genes under multiple abiotic stress conditions

**Figure 7:**
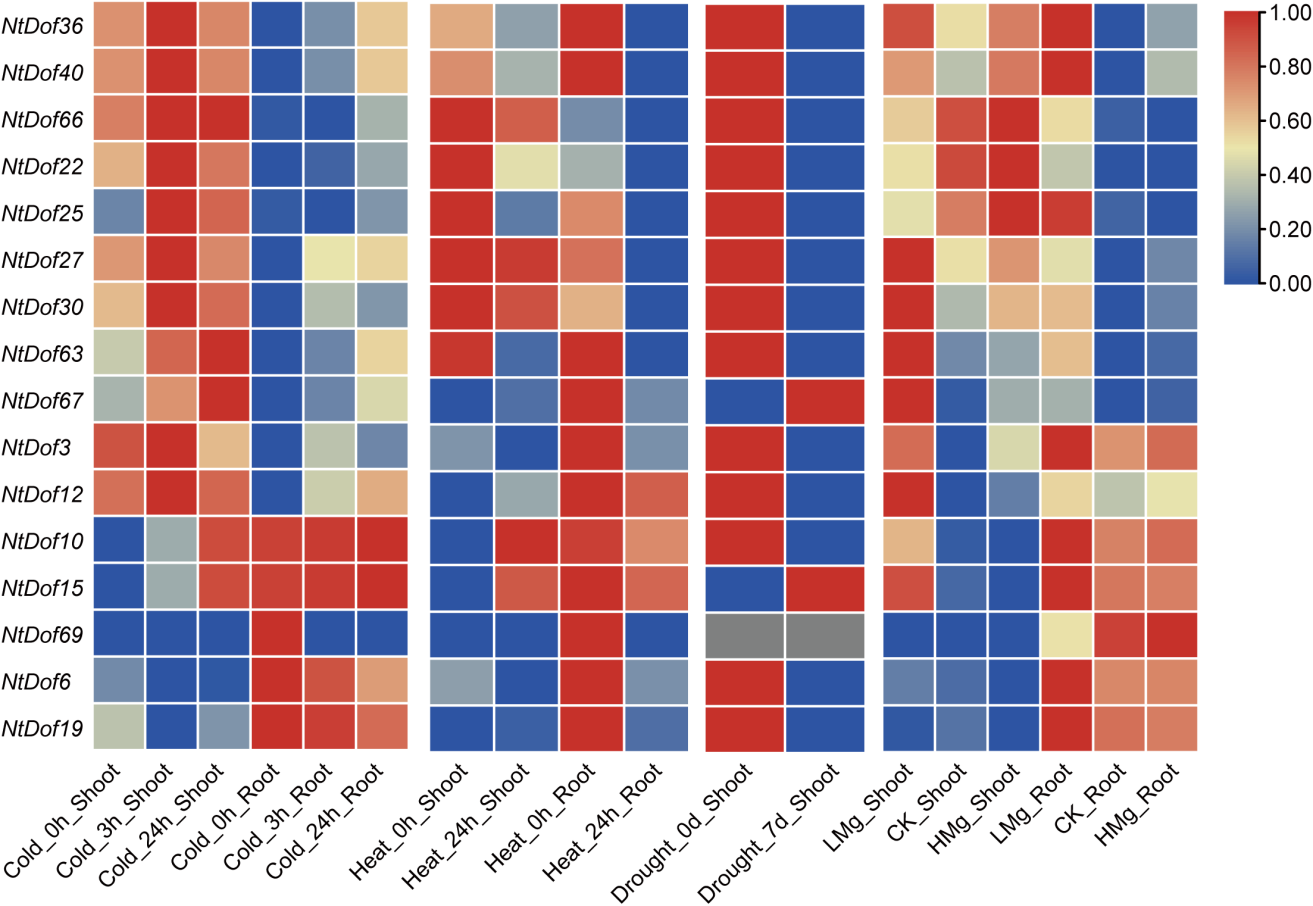
Expression profiles of *NtCDF* family genes in tobacco in response to multiple abiotic stress treatments, including cold (4°C, 24h), heat (45°C, 24h), drought, and magnesium deficiency stress. Heatmaps were generated in TBtools using a row-wise normalization scale from 0 to 1.

To investigate the roles of *CDFs* in responses to diverse abiotic stresses, the expression dynamics of *NtCDF* genes were analyzed using transcriptome datasets previously generated in our laboratory. These datasets included cold stress (3 h and 24 h treatments at 4 °C) (Shao et al., 2024), heat stress (24 h at 45 °C) (Chen et al., 2024), drought stress (7 days of natural drought), and magnesium deficiency stress (Chen et al., 2025). The results showed that the majority of *NtCDF* genes were responsive to low temperature stress (**Figure 7**). However, except *NtDof6*, *NtDof19*, and *NtDof69*, all *NtCDF* members exhibited an early response at 3h following cold stress treatment, followed by reduced expression levels after 24h of cold exposure. Interestingly, most *NtCDFs* displaying this expression pattern were also suppressed under heat stress conditions. In contrast to previous studies implicating drought tolerance in *CDFs*, *NtCDF* genes in tobacco showed little to no response to drought stress. However, nearly all *NtCDFs* except *NtDof22/25/66* responded to magnesium deficiency stress. *NtDof22/25/66* were downregulated under low magnesium conditions, the remaining *NtCDF* genes exhibited upregulated expression in response to magnesium deficiency, indicating a potential role of *NtCDFs* in nutrient stress adaptation.

### *NtCDF* genes differentially regulate tobacco responses to cold stress

**Figure 8:**
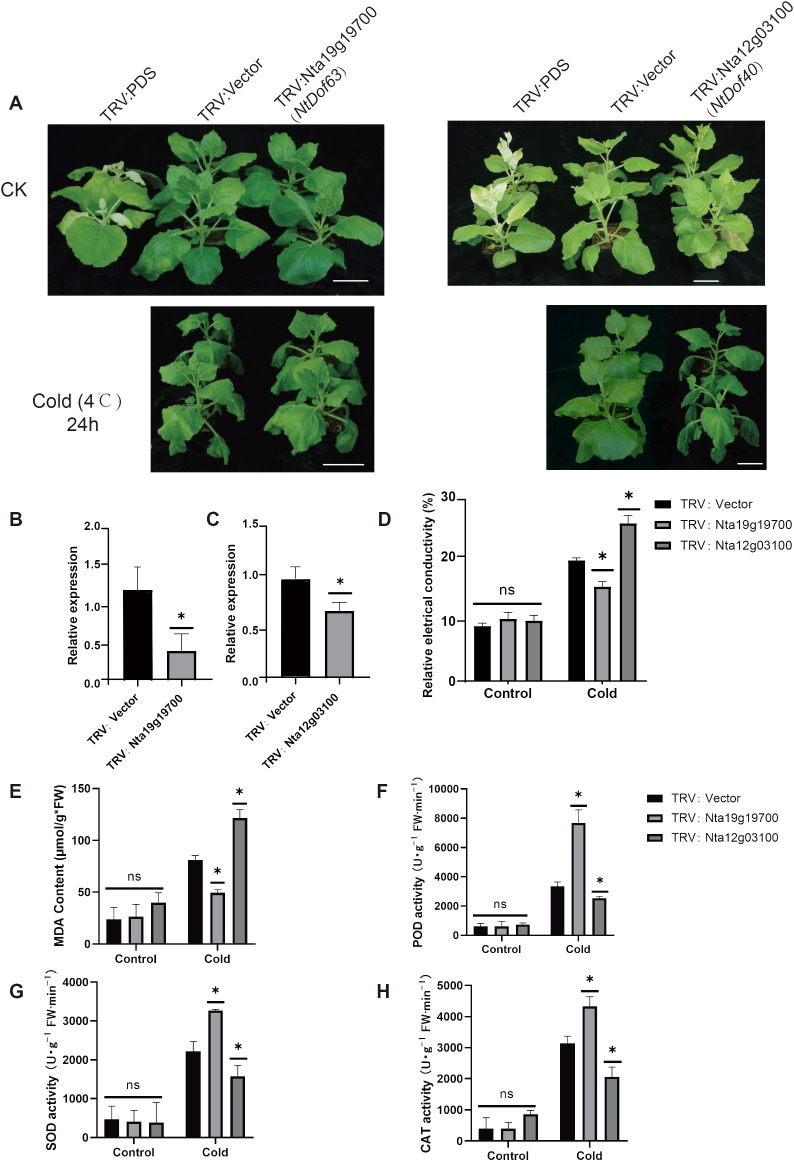
The phenotypic, physiological, and biochemical indicators of cold stress in VIGS *Nicotiana benthamiana* seedlings. **(A)** The control group (25 °C for 24h), cold stress group (4 °C for 24h), Bar=10 cm. Relative expression levels of VIGS-mediated knock-down of gene *NtDof63* **(B)** and *NtDof40* **(C)**. **(D)** Leaf relative conductivity. The malondialdehyde (MDA) content **(E)** and the activities of the antioxidant enzymes peroxidase [POD; **(F)**] superoxide dismutase [SOD; **(G)**], and catalase [CAT; **(H)**] Significance was assessed using more than four biological replicates, with asterisks denoting statistically significant differences between treatments as determined by the paired Student’s *t*-test (*P < 0.05).

Structural characterization of the *NtCDF* subfamily revealed distinct motif architectures that differentiate them from canonical *DOF* proteins, coupled with promoter analyses showing significant enrichment of stress responsive *cis*-regulatory elements in *NtCDF* members. To elucidate the primary function of *NtCDF* in plant stress responses, we performed Virus-Induced Gene Silencing (VIGS). Two representative *NtCDF* genes, *NtDof40* (*Nta19g19700*) and *NtDof63* (*Nta12g03100*) were selected for functional analysis (**Figure 8A**). The results demonstrated a reduction in gene expression levels by 61.45% and 31.59% in the silenced plants, respectively (**Figures 8B, C**). After a one day exposure to cold stress, the *NtDof63* silenced plants showed less leaf wilting compared to the control plants. Physiological indices, including relative electrical conductivity and malondialdehyde (MDA) content, were significantly lower in *NtDof63* silenced plants than the control plants. Whereas the activities of key antioxidant enzymes such as peroxidase (POD), superoxide dismutase (SOD), and catalase (CAT) were markedly increased (**Figures 8D–H**). In contrast, the *NtDof40* silenced plants displayed opposite physiological trends under cold stress including, increased membrane damage and reduced antioxidant enzyme activities. Collectively these findings suggest that *NtDof40* potentially serves as a positive regulator in the tobacco cold stress response, while *NtDof63* appears to function as a negative regulator in cold stress response.

## Discussion

### Evolutionary origin and conserved functions of the plant *Dof* gene family

The *Dof* gene family, a well characterized group within the zinc finger (ZF) superfamily (Yang et al., 2022; Zhang et al., 2022), exhibits a remarkable functional diversity and conservation across plant lineages. Elucidating its evolutionary trajectory is therefore essential for understanding the regulatory mechanisms underlying plant growth and development. In this study, phylogenetic reconstruction across key plant lineages revealed that *Dof* genes first emerged in ferns, coinciding with the origin of vascular systems (**Figure 1**). This evolutionary milestone marks the transition from phycophyta to pteridophyta and underscores a close association between the emergence of Dof transcription factors and vascular tissue development. Recent single-cell sequencing study further supports this evolutionary link by demonstrating that, in both C3 and C4 plants, Dof transcription factors exhibit preferential binding and expression in bundle sheath cells. This conserved pattern activates an ancestrally conserved C3-derived transcriptional network in C4 species, ultimately conferring the unique photosynthetic capabilities that distinguish C4 plants from their C3 counterparts (Swift et al., 2024). Furthermore, accumulating studies have confirmed that the *Dof* family likely functions as key regulators in vascular development. These TFs initiate expression and exert their functions during early vascular system formation, as demonstrated by members such as *AtDof2.1*, *AtDof4.6*, *AtDof5.3*, and *AtDof5.8* (Konishi and Yanagisawa, 2007; Gardiner et al., 2010; Ding et al., 2025). Collectively, the evolutionary history, molecular functions, and functional localization of the *Dof* family TFs illustrate their central and potential roles in the regulation of growth and developmental processes as well as vascular system development.

### Genomewide characterization and functional divergence of the *NtDof* gene family in tobacco

In this study, a total of 75 *NtDof* genes were systematically identified in tobacco and categorized into seven distinct subfamilies (**Figure 3**). The distribution and expansion pattern of these genes differ markedly from those in *Arabidopsis thaliana*, a divergence that is likely attributable to the allopolyploid nature of tobacco genome (Wang et al., 2024). Such genomic complexity provides increased opportunities for gene duplication and subsequent functional diversification. Gene structure and conserved motifs analyses revealed that, with few exceptions, members of the *NtCDF* subfamily (Group II) predominantly possess a conserved “2 exons/1 introns” structure along with a unique combination of motifs (motifs 3, 4, 5, 7) (**Figure 4**). This structural specificity has also been identified in the CDF subfamilies of other species, including tomato and *Arabidopsis thaliana* (Corrales et al., 2014; Xu et al., 2020), indicating a potential structural basis for their functional specialization.

Promoter cis-acting elements analysis indicates that *NtCDF* subfamily are enriched in regulatory elements responsive to abscisic acid (ABA), methyl jasmonate (MeJA), low temperature and drought stress (**Figure 6**). This finding aligns with the central role of the *CDF* subfamily in mediating responses to abiotic stress (Renau-Morata et al., 2017; Jin et al., 2024). Furthermore, the investigation of gene duplication events has identified tandem duplication as the primary mechanism driving the expansion of the tobacco *NtDof* family. This pattern is consistent with the expansion mechanisms observed in the *Dof* gene family in other plant species, such as potato and *Salvia miltiorrhiza* (Venkatesh and Park, 2015; Lv et al., 2025), thereby underscoring the significance of tandem duplication in the functional diversification of transcription factor families and the adaptive evolution of species. In comparison to diploid species like *Arabidopsis thaliana* and tomato, each with 36 *Dof* genes, the *NtDof* gene family in tobacco demonstrates greater diversity. This diversity is hypothesized to provide a crucial genetic foundation for tobacco’s ability to withstand complex environmental stresses while maintaining a balance between growth and development.

### Distinct roles of *NtDofs* genes in regulating cold stress responses

Numerous studies have demonstrated the active involvement of the *Dof* gene family in plant stress responses (Corrales et al., 2014; Xu and Dai, 2016; Renau-Morata et al., 2017). In our investigation, we discovered that *CDF* subfamily members possess distinct characteristics in both gene structure and promoter regions that differentiate them from the broader *Dof* family (**Figure 4**). The roles of the *CDF* subfamily in response to abiotic stress have been extensively documented. For instance, the *CDF3* transcription factor has been shown to enhance biomass and yield in tomatoes under salt stress, improve fruit quality, and participate in the regulation of carbon and nitrogen metabolism (Renau-Morata et al., 2017). In rapeseed, *BnCDF1* is implicated in the regulation of flowering and the response to cold stress (Xu and Dai, 2016). Similarly, *RsCDF3* in carrots is positively involved in the regulation of cold stress response (He et al., 2023). Consequently, we selected *NtDof40* and *NtDof63* from the *NtCDF* subfamily for virus-induced gene silencing (VIGS) experiments. Among these, the *NtDof40* silenced plants exhibited sensitivity to cold stress, akin to the function of *NtCDF3* in tomatoes, whereas the *NtDof63* silenced plants demonstrated tolerance to cold stress. Further assays confirmed this cold adaptation is likely achieved through active modulation of the ROS system, whereas most other *Dof* members lack such stress responsive expression patterns (**Figure 8**). These findings not only yield functional evidence for the specific regulatory role of the CDF subfamily in plant stress adaptation but also clarify that *NtCDF* genes play distinct roles in mediating plant stress responses.

## Conclusion

This study provides a comprehensive evolutionary and functional characterization of the *Dof* transcription factor family in tobacco. Phylogenomic analysis revealed that *Dof* genes originated early in plant evolution and expanded extensively in angiosperms, largely driven by gene duplication. A total of 75 *NtDof* genes were identified and classified into seven subfamilies, with the *NtCDF* clade showing conserved gene structures, unique motif compositions, and enrichment of stress and hormone responsive *cis*-elements. Expression profiling demonstrated that *NtCDF* genes respond dynamically to abiotic stresses, particularly cold and magnesium deficiency. Functional validation using virus-induced gene silencing confirmed that *NtCDF* members play divergent roles in cold stress tolerance, with *NtDof40* acting as a positive regulator and *NtDof63* functioning as a negative regulator. Together, these findings highlight the evolutionary diversification and functional importance of *NtCDF* genes in tobacco stress adaptation.

## Data Availability

The original contributions presented in the study are included in the article/**Supplementary Material**. Further inquiries can be directed to the corresponding authors.
